# Commentary: Bacteriophage transfer during faecal microbiota transplantation in *Clostridium difficile* infection is associated with treatment outcome

**DOI:** 10.3389/fcimb.2018.00104

**Published:** 2018-04-04

**Authors:** Blessing O. Anonye

**Affiliations:** ^1^Microbiology and Infection Unit, Division of Biomedical Sciences, Warwick Medical School, Coventry, United Kingdom; ^2^Warwick Integrative Synthetic Biology Centre, School of Life Sciences, University of Warwick, Coventry, United Kingdom

**Keywords:** bacteriophages, gut microbiota, fecal microbiota transplantation, *C. difficile* infection, *Caudovirales*

Fecal microbiota transplantation (FMT) has been used as a treatmentof last resort for recurrent *C. difficile* infections (CDI) with a cure rate of 85–90% after the first FMT (van Nood et al., 2013; Jiang et al., 2017). Several studies have examined the changes that occur in the bacterial community that leads to reestablishment of the intestinal microbiota (Fuentes et al., 2014; Seekatz et al., 2014; Staley et al., 2016). However, studies that have investigated the role of viruses in FMT are limited (Broecker et al., 2016a,b, 2017; Ott et al., 2017).

Recently, Zuo and colleagues performed metagenomics sequencing of virus like particles on fecal samples from patients with CDI and healthy controls to determine if bacteriophages were associated with restoration of the intestinal microbiota after FMT (Zuo et al., 2017). Prior to FMT, the patients had increased abundance of *Caudovirales* with decreased diversity, richness and evenness when compared to healthy controls. Longitudinal studies of patients who received FMT (*n* = 9) and standard therapy, vancomycin (*n* = 5) demonstrated that FMT led to the transfer of viruses from the donor to the recipients (Zuo et al., 2017).

The patients that were administered FMT were divided into two groups of “responders” and “non-responders” based on whether they were cured of CDI or recurred after FMT. In particular, after FMT, they noticed a significant decrease in the abundance of *Caudovirales* and increase in richness of donor-derived *Caudovirales* in the enteric virome of the patients (Zuo et al., 2017). There was a correlation between donor viral richness and the patients responding to FMT. Of the two-thirds that were cured after FMT, four of the donors had higher *Caudovirales* richness when compared to the non-responders group where the donor *Caudovirales* richness was lower (Zuo et al., 2017). When compared to the non-responders, the remaining two donors had a similar (*n* = 1 for non-responder donor) or slightly higher *Caudovirale* richness.

Furthermore, lower abundance of the family, *Microviridae* was observed in the patients before FMT when compared to the controls but this increased after FMT. Fifteen viral species were found to be enriched between FMT responders and non-responders. Viral species belonging to the *Microviridae* family such as *Eel River Basin pequenovirus* was the most abundant in the responders (Zuo et al., 2017).

Moreover, Zuo et al. performed 16S rRNA gene sequencing to determine changes in the bacterial community and noted increase in *Lachnospiraceae* and *Ruminococcaceae* families (Zuo et al., 2017). However, there was no significant difference between donor transferred bacteria between FMT responders and non-responders. Interestingly, vancomycin treatment had no significant effect on the viral community (*Caudovirales*) of those who responded to the antibiotic therapy. However, the bacterial community was significantly affected (Zuo et al., 2017).

Broecker et al. investigated the long term bacterial and virome changes in a patient after FMT for recurrent CDI, and found the virome at several months post-FMT related to the donor virome (Broecker et al., 2016a,b). Similarly, Ott and colleagues recently demonstrated that sterile fecal filtrates from donor feces was effective in treating recurrent CDI in five patients (Ott et al., 2017). They showed that the “phagebiota” of a recipient at 6 weeks post-FMT was similar to the fecal filtrate from the donor (Ott et al., 2017). These findings indicate that apart from live bacteria, other components of the microbiota such as bacteriophages, antimicrobial compounds or metabolites contribute to reestablishment of the intestinal microbiota in FMT.

There is no doubt that bacteriophages play a role in the intestinal microbiota with a potential to alter the composition and function of the host microbiota. The question is what constitutes a healthy gut phageome and how do they influence the human gut microbiota? Most of the phages in healthy human gut microbiota belong to the *Caudovirales* order and from the family *Microviridae* which have double and single stranded DNA respectively (Kim et al., 2011; Manrique et al., 2016). As seen above in recurrent CDI, increased diversity, richness and evenness of the *Caudovirales* was implicated in the efficacy of FMT. However, in other intestinal diseases, it is not clear cut as to the role of *Caudovirales* likely due to other risk factors involved (Norman et al., 2015). For example, *Caudovirales* richness was observed in patients with inflammatory bowel disease (Norman et al., 2015).

The next question one may be tempted to ask is, how stable is the “phagebiota” in individuals and what are the effects of antibiotic perturbations on the phageome? Research by Ly et al. showed that transmission of viruses was common between members of the same household over a 6-month period (Ly et al., 2016). Treatment of healthy individuals living in a particular household with the antibiotics, amoxicillin or azithromycin for 7 days did not affect the composition of viruses in the intestinal microbiota (Ly et al., 2016).

These studies have highlighted the underappreciated role viruses play in addition to bacterial colonization in the intestinal microbiota. However, as individual phages are specific in action toward their bacterial host, it would be advantageous to isolate phages that target pathogenic bacteria such as *C. difficile*, though this is not trivial. Indeed, previous work revealed that using a single phage, ΦCD27 (Meader et al., 2010, 2013) or a combination of phages led to the inhibition of *C. difficile* growth *in vitro* and *in vivo* (Nale et al., 2016a,b). Recently, a combination of four phages was found to totally inhibit *C. difficile* growth in a batch fermentation model spiked with feces from four healthy volunteers (Nale et al., 2018).

Much work remains to be done on phage therapy for *C. difficile* infection. The ability to develop a synthetic mixture of phages as treatment for infectious diseases, will go a long way in this era of antibiotic resistance. Not only will this be beneficial in severe CDI, but could also be useful as a therapy for other diseases related to the intestinal microbiota.

## Author contributions

The author confirms being the sole contributor of this work and approved it for publication.

### Conflict of interest statement

The author declares that the research was conducted in the absence of any commercial or financial relationships that could be construed as a potential conflict of interest.

## References

[B1] Broecker F. Klumpp J. Moelling K. (2016a). Long-term microbiota and virome in a Zürich patient after fecal transplantation against *Clostridium difficile* infection. Ann. N. Y. Acad. Sci. 1372, 29–41. 10.1111/nyas.13100 27286042

[B2] Broecker F. Klumpp J. Schuppler M. Russo G. Biedermann L. Hombach M.. (2016b). Long-term changes of bacterial and viral compositions in the intestine of a recovered *Clostridium difficile* patient after fecal microbiota transplantation. Cold Spring Harb. Mol. Case Stud. 2:a000448. 10.1101/mcs.a000448 27148577 PMC4849847

[B3] Broecker F. Russo G. Klumpp J. Moelling K. (2017). Stable core virome despite variable microbiome after fecal transfer. Gut Microbes 8, 214–220. 10.1080/19490976.2016.1265196 27935413 PMC5479397

[B4] Fuentes S. van Nood E. Tims S. Heikamp-de Jong I. ter Braak C. J. Keller J. J.. (2014). Reset of a critically disturbed microbial ecosystem: faecal transplant in recurrent *Clostridium difficile* infection. ISME J. 8, 1621–1633. 10.1038/ismej.2014.13 24577353 PMC4817604

[B5] Jiang Z. D. Ajami N. J. Petrosino J. F. Jun G. Hanis C. L. Shah M. (2017). Randomised clinical trial: faecal microbiota transplantation for recurrent *Clostridum difficile* infection – fresh, or frozen, or lyophilised microbiota from a small pool of healthy donors delivered by colonoscopy. Aliment. Pharmacol. Ther. 45, 899–908. 10.1111/apt.13969 28220514

[B6] Kim M. S. Park E. J. Roh S. W. Bae J. W. (2011). Diversity and abundance of single-stranded DNA viruses in human feces. Appl. Environ. Microbiol. 77, 8062–8070. 10.1128/aem.06331-11 21948823 PMC3208976

[B7] Ly M. Jones M. B. Abeles S. R. Santiago-Rodriguez T. M. Gao J. Chan I. C.. (2016). Transmission of viruses via our microbiomes. Microbiome 4, 64. 10.1186/s40168-016-0212-z 27912785 PMC5134127

[B8] Manrique P. Bolduc B. Walk S. T. van der Oost J. de Vos W. M. Young M. J. (2016). Healthy human gut phageome. Proc. Natl. Acad. Sci. U.S.A. 113, 10400–10405. 10.1073/pnas.1601060113 27573828 PMC5027468

[B9] Meader E. Mayer M. J. Gasson M. J. Steverding D. Carding S. R. Narbad A. (2010). Bacteriophage treatment significantly reduces viable Clostridium difficile and prevents toxin production in an *in vitro* model system. Anaerobe 16, 549–554. 10.1016/j.anaerobe.2010.08.006 20816997

[B10] Meader E. Mayer M. J. Steverding D. Carding S. R. Narbad A. (2013). Evaluation of bacteriophage therapy to control *Clostridium difficile* and toxin production in an *in vitro* human colon model system. Anaerobe 22, 25–30. 10.1016/j.anaerobe.2013.05.001 23685029

[B11] Nale J. Y. Chutia M. Carr P. Hickenbotham P. T. Clokie M. R. (2016a). ‘Get in early’; biofilm and Wax Moth (*Galleria mellonella*) models reveal new insights into the therapeutic potential of *Clostridium difficile* bacteriophages. Front. Microbiol. 7:1383. 10.3389/fmicb.2016.01383 27630633 PMC5005339

[B12] Nale J. Y. Redgwell T. A. Millard A. Clokie M. R. J. (2018). Efficacy of an optimised bacteriophage cocktail to clear *Clostridium difficile* in a batch fermentation model. Antibiotics 7:13. 10.3390/antibiotics7010013 29438355 PMC5872124

[B13] Nale J. Y. Spencer J. Hargreaves K. R. Buckley A. M. Trzepinski P. Douce G. R.. (2016b). Bacteriophage combinations significantly reduce clostridium difficile growth *in vitro* and proliferation *in vivo*. Antimicrob. Agents Chemother. 60, 968–981. 10.1128/aac.01774-15 26643348 PMC4750681

[B14] Norman J. M. Handley S. A. Baldridge M. T. Droit L. Liu C. Y. Keller B. C.. (2015). Disease-specific Alterations in the enteric virome in inflammatory bowel disease. Cell 160, 447–460. 10.1016/j.cell.2015.01.002 25619688 PMC4312520

[B15] Ott S. J. Waetzig G. H. Rehman A. Moltzau-Anderson J. Bharti R. Grasis J. A.. (2017). Efficacy of sterile fecal filtrate transfer for treating patients with *Clostridium difficile* Infection. Gastroenterology 152, 799.e797–811.e797. 10.1053/j.gastro.2016.11.010 27866880

[B16] Seekatz A. M. Aas J. Gessert C. E. Rubin T. A. Saman D. M. Bakken J. S.. (2014). Recovery of the gut microbiome following fecal microbiota transplantation. MBio 5:e00893-14. 10.1128/mBio.00893-14 24939885 PMC4068257

[B17] Staley C. Kelly C. R. Brandt L. J. Khoruts A. Sadowsky M. J. (2016). Complete microbiota engraftment is not essential for recovery from recurrent *Clostridium difficile* infection following fecal microbiota transplantation. MBio 7:e01965-16. 10.1128/mBio.01965-16 27999162 PMC5181777

[B18] van Nood E. Vrieze A. Nieuwdorp M. Fuentes S. Zoetendal E. G. de Vos W. M.. (2013). Duodenal infusion of donor feces for recurrent *Clostridium difficile*. N. Engl. J. Med. 368, 407–415. 10.1056/NEJMoa1205037 23323867

[B19] Zuo T. Wong S. H. Lam K. Lui R. Cheung K. Tang W.. (2017). Bacteriophage transfer during faecal microbiota transplantation in *Clostridium difficile* infection is associated with treatment outcome. Gut 67, 634–643. 10.1136/gutjnl-2017-313952 28539351 PMC5868238

